# Epidemiological and clinical features, response to HAART, and survival in HIV-infected patients diagnosed at the age of 50 or more

**DOI:** 10.1186/1471-2334-6-159

**Published:** 2006-11-06

**Authors:** MaMercedes Nogueras, Gemma Navarro, Esperança Antón, Montserrat Sala, Manel Cervantes, MaJosé Amengual, Ferran Segura

**Affiliations:** 1 Infectious Diseases Program, Corporació Sanitària Parc Taulí, Sabadell, Barcelona, Spain; 2 UDIAT Diagnosis Centre, Corporació Sanitària Parc Taulí, Sabadell, Barcelona, Spain

## Abstract

**Background:**

Over the last years, the mean age of subjects with HIV infection and AIDS is increasing. Moreover, some epidemiological and clinical differences between younger and older HIV-infected individuals have been observed. However, since introduction of HAART therapy, there are controversial results regarding their response to HAART. The aim of the present study is to evaluate epidemiological and clinical features, response to HAART, and survival in elderly HIV-infected patients with regard to younger HIV-infected patients.

**Methods:**

A prospective cohort study (1998–2003) was performed on patients from Sabadell Hospital, in Northeast of Spain. The cohort includes newly attended HIV-infected patients since January 1, 1998. For the purpose of this analysis, data was censured at December 31, 2003. Taking into account age at time of diagnosis, it was considered 36 HIV-positive people aged 50 years or more (Group 1, G1) and 419 HIV-positive people aged 13–40 years (Group 2, G2). Epidemiological, clinical, biological and therapy data are recorded. Statistical analysis was performed using Chi-squared test and Fisher exact test, Mann-Whitney U test, Kaplan-Meier, Log Rank test, and Two-Way ANOVA from random factors.

**Results:**

G1 showed higher proportion of men than G2. The most common risk factors in G1 were heterosexual transmission (*P *= 0.01) and having sex with men or women (*P *< 0.001). G1 and G2 show parallel profiles through the time regarding immunological response (*P *= 0.989) and virological response (*P *= 0.074). However, older people showed lower CD4 cell counts at first clinic visit (*P *< 0.001) and, eventually, they did not achieve the same counts as G2. G1 presented faster progression to AIDS (*P *< 0.001) and shorter survival (*P *< 0.001).

**Conclusion:**

Older patients have different epidemiological features. Their immunological and virological responses are good. However, older patients do not achieve the same CD4 cell counts likely due to they have lower counts at first clinic visit. Thus, it is essential physicians know older HIV-infected patients features to consider the possibility of HIV infection in these patients with the aim of treatment would not be delayed.

## Background

Over the last years, the proportion of old individuals infected with HIV is increasing. For instance, patients older than 50 years were more than 10 % of AIDS notifications made in the last ten years in USA [1]. More than 90 500 individuals over 50 years are living with AIDS in this country [2]. In Europe, 12.6 % of AIDS patients were older than 50 years in 1998, whereas 14.5 % of them had more than 50 years in 2002 [3]. Since the beginning of the HIV pandemic to 2005, 71 039 cases of AIDS have been reported in Spain. Mean age at time of diagnosis was 40.3 years [4].

The progressive increase of mean age of subjects with HIV infection and AIDS could be explained by different factors. Since 1996, when highly active antiretroviral therapy (HAART) was available, the history of HIV infection has evolved from acute or subacute disease to a chronic and controllable infection. Thus, HIV-infected people grow older and live longer. Antimicrobial therapy and chemoprophylaxis have also allowed the increase survival of people living with HIV infection. Moreover, some epidemiological features have changed. The importance of sexual transmission as mode of HIV spread is increasing [1]. Therefore, there are a number of patients who are not aware of their infection, delaying the diagnosis.

There are epidemiological differences between younger and older HIV-infected individuals. Older people have a higher prevalence of sexual HIV transmission, a reduction of transmission due to intravenous use, and a higher proportion of men [5,6]. On the other hand, there are controversial data about the virological and immunological responses to HAART as well as survival in older patients. Some studies indicates that the duration of survival is significantly shorter for elderly people due to deficiencies in the immune system related to age [7,8]. However, others studies seem to show older people have the same response to HAART than younger patients [9,10].

In the past, there was little attention to older people with HIV infection due to the little number of them. Nevertheless, the importance of knowledge about the HIV infection in this population is increasing. The aim of the present study is to characterize the ways in which older HIV infected people differ from younger, in a Spanish cohort. Our previously hypothesis are: firstly, older patients have different epidemiological and clinical features; secondly, they response to HAART in the same way that younger patients. The survey takes into account epidemiological and clinical features, virological and immunological responses, prognostic, and survival.

## Methods

### Study setting

Subjects were enrolled at the Sabadell Hospital. This is a 765-bed general teaching hospital located in Barcelona, Spain. It provides primary and specialty care for HIV infection to 850 patients, approximately.

### Design

A prospective cohort study (1998–2003)

### Database

Eligible patients were enrolled at the PISCIS project. The latter is a multicenter cohort (10 hospitals of Catalonia and one from Balearic Islands) of HIV-infected people whose features are entered into a computerized database [11]. Inclusion criteria are: to be more than 16 years old, to have confirmed positive serology against HIV, and to be attended at first time from January 1, 1998 in one hospital of the study. All patients with these conditions are included in PISCIS cohort independently of AIDS criteria. Characteristics at first clinic visit are recorded for all patients by clinicians. Epidemiological, clinical, biological and antiretroviral therapy data are sent to the Coordination Centre every four months. Patients' names were codified to attain anonymity. The project was conducted under the terms of Ethics committee approval from Sabadell Hospital.

### Study population

Five hundreds twelve subjects from Sabadell's Hospital included in the PISCIS project were analyzed. They were enrolled between January 1, 1998, and December 31, 2003. Two groups were defined taking into account the age at the time of diagnosis. Patients were included in two age groups. Group 1 (G1) consisted of HIV-infected people 50 years or older (n = 36), whereas Group 2 (G2) consisted of patients aged 13 – 40 years (n = 419). Like other studies [9,10,12-15]], patients aged between G1 and G2 groups (n = 57) were excluded in order to separate both groups. It was decided that the cut off would be 50 years old considering many previously HIV-infection studies data as well as most significant immunological change take place around this age.

### Parameters evaluated

For each subject enrolled, the following data were obtained: age, gender, HIV-risk behavior, data of the first available HIV test, CDC stage of HIV infection, date of AIDS diagnosis, number of visits, cause of visit, number of hospitalizations after HIV diagnosis, antiretroviral therapy, AIDS-defining diseases, and data of death. Data collected from laboratory were: number of CD4+ and CD8+ T cells, and HIV RNA viral load with a limit of detection below 200 copies/ml. Subjects underwent periodical clinical evaluations including laboratory determinations. AIDS criteria were established by Centers for Disease Control and Prevention (CDC), category C.

### Statistical analysis

Software program SPSS 11.5 (SPSS Inc, Chicago IL) was used.

Group comparisons were performed using Chi-squared test. However, if a cell of the table had few expected cases (< 5), Fisher exact test was used.

In baseline characteristics analysis, *P-values *were adjusted by Bonferroni procedure in order to control the familywise Type I error-rate because thirteen simultaneous hypotheses were tested.

All continuous variables were compared by Mann-Whitney U test because their distributions were not normal.

Kaplan-Meier and Log Rank test were used to estimate and to compare survival functions between two groups.

The mean increase in the CD4 cell count and the mean decrease in viral load over time were studied using a mixed ANOVA procedure. Two-Way ANOVA from random factors were used where age group, and time were fixed factors, and patient was random factor. This model studies effect of age, time and interaction between age and time, on CD4 cell count or viral load. Residuals independency and normality were verified.

Patients were recorded as alive at their last visit. A P < 0.05 was considered significant.

## Results

### Study population

**Figure 1:**
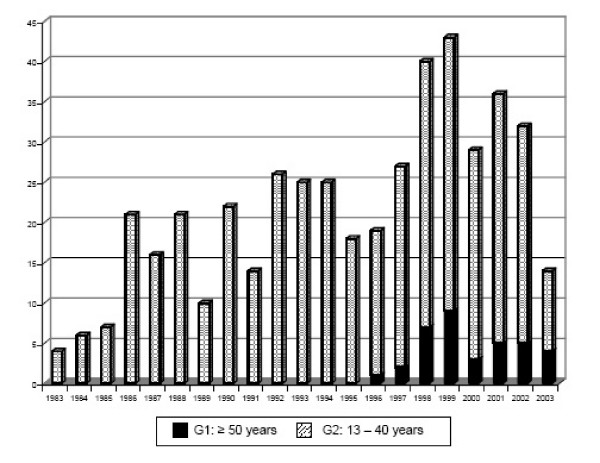
Evolution of HIV diagnosed cases considering age groups.

A total of 512, who were newly attended in our hospital since January 1, 1998, to December 31, 2003, were analyzed. They have been diagnosed of HIV-infection since 1983 to 2003. The mean age of HIV patients at first diagnosis is progressively rising: 23.34 ± 4.33 years (14 – 33 years) at 1983 – 1988 period, 26.32 ± 4.73 years (16 – 36 years) at 1989 – 1993 period, 31.45 ± 9.04 years (15 – 69 years) at 1994 – 1998 period, and 37.27 ± 14.25 years (17 – 82 years) at 1999 – 2003 period. There were no HIV-infected people being 50 years or older at the time of diagnosis until 1996, when the first old patient was enrolled (Fig 1).

### Characteristics at first clinic visit

A total of 36 subjects belonged to G1 (7.9 %) and 419 individuals to G2 (92.1 %). Their characteristics at first clinic visit are shown (Table 1). The mean age was 62.44 ± 9.57 years (50 – 82 years) for the older group and 28.29 ± 6.15 years (14 – 40 years) for the younger group. Older group showed higher proportion of men than younger group.

**Table 1:** Characteristics of the Sample and its Subgroups at first clinic visit

Characteristic	Total Sample	Group 1 (G1)	Group 2 (G2)	*P*-value ¶
Sample size (n)	455	36	419	
Sex: n (%)				
Male	334(73.4)	33 (91.7)	301 (71.8)	0.127
Female	121(26.6)	3 (8.3)	118 (28.2)	
HIV risk factor: n (%)				
IDU *	285(62.9)	2 (5.7)	283 (67.7)	< 0.001
Heterosexual	123(27)	16 (44.4)	107 (25.5)	0.132
MSM **	23(5.1)	4 (11.1)	19 (4.5)	< 0.999 ∥
MSMW ‡	8(1.8)	6 (16.7)	2 (0.5)	< 0.001 ∥
Unknown	16(3.5)	8 (22.2)	8 (2)	< 0.001 ∥
AIDS criteria: n (%)				
Yes §	57(12.5)	7 (19.4)	50 (11.9)	< 0.999 ∥
No	398(87.5)	29 (80.6)	369 (88.1)	
Previous treatment: n (%)				
Yes	181(39.8)	2 (5.6)	179 (42.7)	< 0.001
No	274(60.2)	34 (94.4)	240 (57.3)	
CD4 (n° cel./mm^3^)	352.08 ± 294.10	180.83 ± 230.85	366.90 ± 294.52	< 0.001
CD8 (n° cel./mm^3^)	957.82 ± 597.10	963.38 ± 733.22	956.97 ± 583.73	< 0.999
Plasma viral load (log10)	4.09 ± 1.22	4.51 ± 1.18	4.05 ± 1.22	0.451
HBsAg #: n (%)				
Positive	24(8.1)	1 (4.5)	23 (8.3)	< 0.999
Negative	274(91.9)	21 (95.5)	253 (91.7)	
Anti-HCV: n (%)				
Positive	197(56.8)	3 (13.6)	194 (65.1)	< 0.001
Negative	123(43.2)	19 (86.4)	104 (34.9)	

¶ *P value *adjusted by Bonferroni procedure; * IDU: Injection drug user; ** MSM: Men who have sex with men; ∥ Fisher exact test; ‡ MSMW: Men who have sex with men or women; § AIDS criteria: Centers for Disease Control and Prevention (CDC) category C; # HBsAg: Hepatitis B superficial antigen

The most common risk behavior for the acquisition of HIV infection in G1 was sexual contact, whereas injection drug use was the most important risk factor in G2. Moreover, there were a greater percentage of older patients whose risk behavior is unknown.

At the enrollment, there was no statistically significant difference between both groups with regard to the AIDS criteria. However, time from diagnosis of HIV to enrollment was lower in elderly group (*P *< 0.001). In fact, seven patients from G1 presented AIDS criteria at the enrollment and their mean time since diagnosis of HIV was 6.57 ± 11.93 days (0 – 30 days). Fifty patients from G2 were enrolled with AIDS criteria and their mean time since diagnosis of HIV was 2273.72 ± 2217.03 days (0 – 7376 days).

In the younger group, there were more individuals with previous treatment than in older group (*P *< 0.001).

At first clinic visit, elderly group had a lower mean CD4 cells count [180.83 ± 230.85 (5–1309) cells/mm^3 ^versus 366.89 ± 294.52 (2–1819) cells/mm^3^,], and a higher viral load [4.51 ± 1.18 (2.3–5.88) log_10 _copies/mL versus 4.05 ± 1.22 (1.59–5.97) log_10 _copies/mL]. There were not differences relating to CD8 cells count.

There was higher percentage of younger people who had antibodies against hepatitis C virus (*P *< 0.001).

Elderly group had higher number of patients who were admitted to the hospital (47.2 % versus 30.1 %, *P *= 0.032). Most elderly patients were admitted to the hospital because of *Pneumocystis jiroveci *infection (41.2 %), tuberculosis (11.8 %), *Herpes zoster *infection (5.9 %), and Kaposi's sarcoma (5.9 %). On the other hand, young patients were admitted to the hospital because of widely conditions as *Pneumocystis jiroveci *infection (12 %), tuberculosis (9.6 %), bacterial pneumonia (8 %), hepatic diseases, renal diseases, cardiovascular diseases, encephalopathy, or different kind of infectious diseases.

### Treatment

At the enrollment, there was more percentage of younger patients who received previous treatment. Nevertheless, most patients from both age groups were treated during follow-up. In fact, 31 (86.1 %) of individuals being 50 years or older and 338 (80.7 %) of individuals aged 13–40 years received at least one antiretroviral drug for some time during follow-up. Number of patients from G1 and G2 who were receiving treatment at one, two, three, and four years of follow-up were 23/23 (100%) versus 148/164 (90.2%), 17/17 (100%) versus 111/119 (93.3%), 13/13 (100%) versus 71/75 (94.7%), and 8/8 (100%) versus 46/50 (92%), respectively. There were no statistically significant differences between groups at any point of time.

Whereas there was no difference in the use of antiretroviral, the mean time between HIV diagnosis and therapy initiation is lower in elderly group [3.48 ± 6.35 (0–24.01) months versus 73.11 ± 64.15 (0–240.13) months]. Most of patients received three antiretrovirals (70.9 % in G1 and 61.6 % in G2).

### Immune and virological responses

**Figure 2:**
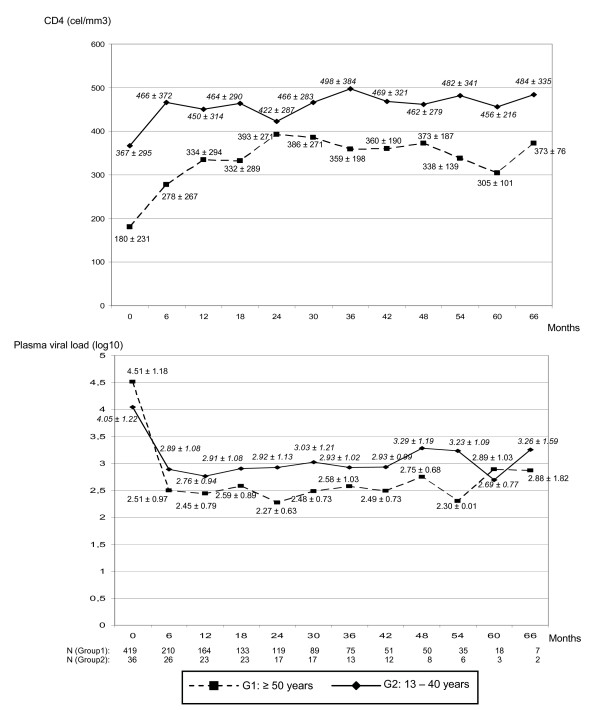
CD4 cell count and viral load (mean ± SD) at first visit and each six months of follow-up.

CD4 cell counts and viral load were obtained every six months during the follow-up since first visit. Figure 2 shows the mean data from CD4 cell counts and viral load according to time and age group. The mixed ANOVA procedure was carried out in order to study if both age groups showed similar virological and immunological responses.

Patients from G1 had higher viral load and lower CD4 cell count at the enrollment. In both groups, greatest immune and virological responses were observed in the first six month. However, these patients did not achieve the same CD4 cell counts than younger individuals.

Considering increase of CD4+ cell number, both age group showed parallel profiles (age group × time: *P *= 0.989). Statistically significant increase through the time was observed in both groups (time: *P *= 0.005). Younger patients had higher number of CD4+ cells during follow-up (age group: *P *< 0.001).

Taking into account decrease in plasma viral load, both age groups show not different behavior (age group × time: *P *= 0.074). A statistically significant decrease through the time was observed in both groups (time: *P *< 0.001), and older patients had lower viral load (age group: *P *= 0.013), since 6 months of follow-up.

### Clinical progression

Even if there were no significant difference regarding AIDS criteria at the enrollment, greater number of older patients developed AIDS. Of patients without AIDS criteria at enrollment, percentage of cases and mean time of progression to first AIDS event were calculated. In fact, 18 (50 %) cases occurred in G1, and 121 (28.87 %) in G2 (*P *= 0.006) during follow-up. Compared by Log Rank test, G1 progression to AIDS was higher than G2 (*P *< 0.001). In a Kaplan-Meier analysis, mean times of progression to AIDS were 57.72 ± 8.91 (40.26 – 75.18) months in G1 and 180.04 ± 5.10 (170.05 – 190.03) months in G2. Younger HIV-infected people had a probability of remaining AIDS-free of 96.36 % and 94.32% at one and two years of HIV diagnosis, respectively. However, older individuals' probabilities were 62.96 % and 58.77 % at the same moments.

The most common AIDS-defining diseases are *Pneumocystis jiroveci *infection (25 % vs. 9.3 %), tuberculosis (8.3 % vs. 14.31 %), Kaposi's sarcoma (5.5 % vs. 0.23 %), Candidiasis (5.5 % vs. 2.38 %), *Cytomegalovirus *infection (2.7 % vs. 0.47 %), caquexia (2.7 % vs. 0.23 %), *Herpes simple *infection (2.7 % vs. 0.23 %), and encephalopathy (2.7 % vs. 1.19 %). There are significant statistically differences regarding *Pneumocystis jiroveci *infection (*P *= 0.008) and Kaposi's sarcoma (*P *= 0.017). There were no differences between both groups with regard to time since HIV diagnosis to *Pneumocystis jiroveci *infection. There was no statistical difference between age groups with reference to number of tuberculosis cases. In a Kaplan-Meier analysis, time of progression to tuberculosis was 6.29 ± 0.53 (5.24 – 7.33) in G1 and 16.05 ± 0.42 (15.23 – 16.86) months (Log Rank test, *P *= 0.037). Patients developed one (82.6 %), two (16.7 %) or three (0.7 %) AIDS-defining diseases at same time. However, there was no significant difference between both age groups.

### Survival

**Figure 3:**
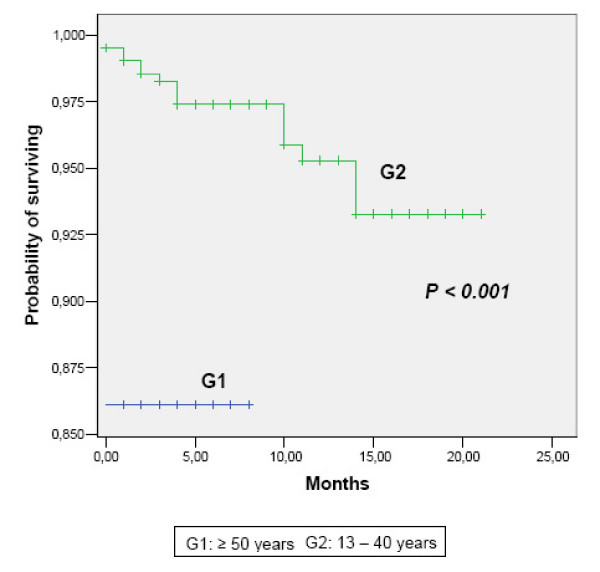
Cumulative survival rate, according to age group.

A total of 16 (3.8 %) patients aged 13–40 years were exitus. In contrast, five (13.9 %) of HIV-infected patients being 50 years or older died during the follow-up (*P *= 0.019). Mean survival time was 2520.6 ± 166.2 (2194.8 – 2846.3) months in G1 and 7343.8 ± 79.9 (7186.9 – 7500.5) months in G2. In figure 3, Kaplan-Meier survival curves are showed. Log Rank test was statistically significant (*P *< 0.001). Every death in the older group occurred in the first year since HIV diagnosis. Cumulative proportions of younger patients who survived were 99.52 % at one year, 98.28 % at three years, and 97.40 % at five years.

## Discussion

Since 1996, when HAART became widely available, elderly patients infected with HIV are a growing population [3,6]. HAART is effective in increasing CD4 cell counts and in decreasing the virus load, so it has been associated with a 50% decrease in morbidity and mortality with AIDS. Therefore, HIV-infected individuals have prolonged survival and they enjoy good life conditions for longer [6]. Besides, the proportion of new infected patients being older 50 years is increasing. In fact, the mean age at the time of diagnosis is increasing in our cohort.

There are epidemiological differences associated with age. Like other surveys [2,5,6,16], older patients in our cohort have a higher prevalence of sexual HIV transmission, a lower risk of transmission due to intravenous use, and a greater proportion of men. Remarkably, the number of older individuals whose risk behavior was unknown was much higher than the number of younger patients.

Unlike old patients, younger ones had higher prevalence of antibodies against Hepatitis C virus. This fact could be due to injected drugs are an important mode of Hepatitis C spread, besides infection drug user is the most prevalent HIV risk factor in younger individuals.

Even though there was more percentage of younger patients who received previous treatment at enrollment, most patients from both age groups received treatment during follow-up. In fact, there were no statistically significant differences between groups at any point of time considering number of patients receiving therapy. Besides, there was no difference in the use of antiretroviral therapy as described in other studies [10,17]. However, after HIV-infection diagnosis, older patients initiated HAART therapy sooner probably due to they are diagnosed later. Interestingly, some studies also described that younger and older patients present the same tolerance to therapy [1,18]. Thus, further studies should be necessary to analyze this fact in our region.

Both age groups show increasing CD4 cell counts and decreasing plasma viral load during follow-up. Like Grabar et al. observations [5], greatest responses were observed in the first six month. Virological response is good in elderly group and they achieve lower viral load. Previous studies showed younger age is associated with lower virological response and virological rebounds [5,12,19,20], probably due to older patients demonstrated better medication adherence. At least 90–95% of adherence is required to achieve optimal treatment [21]. Moreover, an inadequate adherence are also associated with the developed of drug-resistant HIV strains [22] Even though some studies show older age is associated to better adherence [21,23,24], other ones observed that lower virological response in younger age persisted after adjusting for regimen adherence, antiretroviral therapy and disease stage [12,20]. Therefore, others variables could be implied. Adherence information was not surveyed in our database.

The age has been associated to deficiencies in the immune system, with a progressive depletion of lymphocytes [7,17,25]. Moreover, thymic volume, which decreases with age, is associated with recovery of CD4 T cells [1,26]. However, Virad et al. showed CD4 recovery in older patients [13]. Recent data suggest substantial output of CD4 cells can be maintained in advanced age, even with less thymic function. Thus, HAART therapy contributes to immune recovery in older patients, too [10]. At first clinic visit, CD4 cell counts were lower in our older patients. However, both age groups had favorable and parallel immune response. Elderly group did not achieve the same immune restoration, probably due to their lower baseline CD4 cell counts.

Until the introduction of HAART, age was a strong predictor of disease progression and mortality risk [27]. Nevertheless, there are controversial results since 1996. Some studies show that older patients have poorer immune response [7,8]. In contrast, some surveys seem to demonstrate that virological and immune response in older HIV-infected people receiving HAART is similar to that of younger patients [9,14,28,29]. For instance, Perez et al. observed older patients have a great benefit associated with HAART, suggesting lower survival in older people was due to late diagnosis [6]. Tumbarello et al. have demonstrated that older patients under HAART therapy can achieve the same response, although they present a more severe HIV infection [10]. According to our results, it is felt that our analysis agrees with the last studies.

There was no significant difference with regard to AIDS criteria at the enrollment in our patients. Nevertheless, not only greater number of older patients developed AIDS, but also disease progression and mortality since HIV diagnosis were faster in older patients.

Previous data showed that elderly patients are diagnosed later [30,31]]. Taking into account good immunological and virological responses were observed in both groups, faster progression to AIDS and shorter survival in older patients may be due to delayed diagnosis and thus a smaller chance of having received antiretrovirals. In fact, there was lower proportion of elderly patients being on treatment at enrollment. Moreover, elderly patients had lower CD4 cell counts at enrollment and thus they did not achieve the same counts as younger patients at the end of follow-up. There is not a high HIV-infection suspicious in old patients' diagnosis because of: firstly, different epidemiological features associated to age; secondly, the fact that HIV-infection has been associating to younger age. Thus, many old people are unaware of their serostatus. In fact, in our cohort there were a high number of older HIV-infected patients whose risk behavior is unknown. Consequently, even though older patients' response to HAART is parallel to younger patients' response, their immune and virological data were worst at first clinic visit and thus their progression to AIDS was faster and their survival was shorter.

## Conclusion

In conclusion, there are epidemiological differences related to age. Immunological and virological behaviors during follow-up are similar within both age groups. However, older patients have worse features at first clinic visit. Consequently, it is essential physicians know older HIV-infected patients features to consider the possibility of HIV infection in these patients with the aim of treatment would not be delayed.

## Competing interests

*M^a^Mercedes Nogueras, Gemma Navarro, Esperança Antón, Montserrat Sala, Manel Cervantes, M^a^José Amengual*: The authors declare that they have no competing interests.

*Ferran Segura*: He has participated in scientific meeting receiving financial support from pharmaceutical industry regarding antibiotics and antiretrovirals. However, none of them are concerning this study.

## Authors' contributions

MMN carried out the analysis and interpretation of data, and the preparation and revision of manuscript.

GN participated in the study concept and design, and revision of manuscript.

EA participated in the acquisition of subjects and epidemiological and clinical data.

MS participated in the study concept, acquisition of subjects and epidemiological and clinical data.

MC participated in the study concept, acquisition of subjects and epidemiological and clinical data.

MJA participated in the acquisition of data from laboratory.

FS participated in the study concept and design, and revision of manuscript.

## Pre-publication history

The pre-publication history for this paper can be accessed here:


